# Diacetoxyscirpenol, a *Fusarium* exometabolite, prevents efficiently the incidence of the parasitic weed *Striga hermonthica*

**DOI:** 10.1186/s12870-022-03471-6

**Published:** 2022-02-24

**Authors:** Williams Oyifioda Anteyi, Iris Klaiber, Frank Rasche

**Affiliations:** 1 Institute of Agricultural Sciences in the Tropics (Hans-Ruthenberg-Institute), University of Hohenheim, 70593 Stuttgart, Germany; 2 Core Facility Hohenheim, University of Hohenheim, 70593 Stuttgart, Germany

**Keywords:** *Striga hermonthica*, *Fusarium oxysporum* f. sp. *strigae*, *Fusarium* exometabolites, Diacetoxyscirpenol, Biopesticides, Targeted metabolomics, Metabolic footprinting, Trichothecene gene expression

## Abstract

**Background:**

Certain *Fusarium* exometabolites have been reported to inhibit seed germination of the cereal-parasitizing witchweed, *Striga hermonthica*, *in vitro*. However, it is unknown if these exometabolites will consistently prevent *S. hermonthica* incidence *in planta*. The study screened a selection of known, highly phytotoxic *Fusarium* exometabolites, in identifying the most potent/efficient candidate (i.e., having the greatest effect at minimal concentration) to completely hinder *S. hermonthica* seed germination *in vitro* and incidence *in planta*, without affecting the host crop development and yield.

**Results:**

*In vitro* germination assays of the tested *Fusarium* exometabolites (i.e., 1,4-naphthoquinone, equisetin, fusaric acid, hymeglusin, neosolaniol (Neo), T-2 toxin (T-2) and diacetoxyscirpenol (DAS)) as pre-*Striga* seed conditioning treatments at 1, 5, 10, 20, 50 and 100 µM, revealed that only DAS, out of all tested exometabolites, completely inhibited *S. hermonthica* seed germination at each concentration. It was followed by T-2 and Neo, as from 10 to 20 µM respectively. The remaining exometabolites reduced *S. hermonthica* seed germination as from 20 µM (*P* < 0. 0001). *In planta* assessment (in a *S. hermonthica*-sorghum parasitic system) of the exometabolites at 20 µM showed that, although, none of the tested exometabolites affected sorghum aboveground dry biomass (*P* > 0.05), only DAS completely prevented *S. hermonthica* incidence. Following a 14-d incubation of DAS in the planting soil substrate, bacterial 16S ribosomal RNA (rRNA) and fungal 18S rRNA gene copy numbers of the soil microbial community were enhanced; which coincided with complete degradation of DAS in the substrate. Metabolic footprinting revealed that the *S. hermonthica* mycoherbicidal agent, *Fusarium oxysporum* f. sp. *strigae* (isolates Foxy-2, FK3), did not produce DAS; a discovery that corresponded with underexpression of key genes (Tri5, Tri4) necessary for *Fusarium* trichothecene biosynthesis (*P* < 0.0001).

**Conclusions:**

Among the tested *Fusarium* exometabolites, DAS exhibited the most promising herbicidal potential against *S. hermonthica*. Thus, it could serve as a new biocontrol agent for efficient *S. hermonthica* management. Further examination of DAS specific mode of action against the target weed *S. hermonthica* at low concentrations (≤ 20 µM), as opposed to non-target soil organisms, is required.

**Supplementary Information:**

The online version contains supplementary material available at 10.1186/s12870-022-03471-6.

## Background

Cereal production in sub-Saharan Africa (SSA), like many crops in tropical/subtropical agroecosystems, is challenged by various biotic, abiotic, socio-cultural and socio-economic factors [4, 59]. In terms of biotic constraints, the obligate hemiparasitic witchweed, *Striga* spp., especially *Striga hermonthica* (Delile) Benth., is a major factor [19, 54]. According to Gressel et al. [23], out of the 26.23 million ha of all crop fields that were infested by *Striga* spp. in SSA, sorghum fields alone accounted for roughly 20 million ha, thereby causing an estimated yield loss of 6.5 to 6.9 million t per annum. Infestation by *S. hermonthica* begins after the release of organic stimulants (i.e., strigolactones) by the host cereal plants, inducing germination of preconditioned *S. hermonthica* seeds. Then, the elongating radicle of the *S. hermonthica* seedling develops into a haustorium, which is used to attach and penetrate the host cereal root [60]. Successful parasitism of the host plant by *S. hermonthica* is ensured by vascular continuity between the parasite and the host. Through this channel, *S. hermonthica* accesses the host resources to support its life cycle, while the host plant health deteriorates (until death in serious cases), owing mainly to water and nutrient deficiency [66].

*Fusarium oxysporum* f. sp. *strigae* (*Fos*) is a well-known bioherbicide (mycoherbicide), that has proven effective for controlling parasitic *Striga* spp., under both natural and artificial environments [52, 58, 64]. Although, various *Fos* isolates with significant pathogenicity towards *S. hermonthica* have been reported [82], in some cases, the bioherbicidal effectiveness of *Fos* isolates against *S. hermonthica* has shown to be inconsistent [6, 7, 76]. The inconsistent effectiveness of *Fos* isolates against differing *S. hermonthica* populations is, amongst others, a major challenge that deters the widespread application of any bioherbicide against its target weed [18, 26]. Like every functioning ecosystem (natural or artificial), the *Fos*–*S. hermonthica*–cereal crop pathosystem is made up of complex networks of bipartite, tripartite or multipartite interactions that occur both within and between the biotic (cereal crop, *S. hermonthica*, microorganisms) and abiotic (climate, water, soil physico-chemistry) components [48, 53]. These ecologically complex interactions may eventually reduce or in some cases enhance the efficacy of mycoherbicides against their target weeds [38]. However, with respect to the bipartite interaction between *Fos* and *S. hermonthica*, the role of genetic diversity as an underlying factor for this inconsistent effectiveness has been well studied. For instance, the classification of genetically contrasting *Fos* isolates (e.g., Foxy-2 and FK3) into separate vegetative compatibility groups (VCG) and mating types [13], and population genomic structure in *S. hermonthica* populations with varying susceptibility response to Foxy-2 and FK3 [5]. As a result, variation in the metabolome between genetically structured *S. hermonthica* groups were revealed through the sequenced genomic regions of the associated loci to show the differences in the protein-coding nucleotide sequences [5]. Meanwhile, in *Fos*, metabolomic studies have been mainly limited to the detection of particular secondary metabolites that are produced by the fungi and released into culture media e.g., fusaric acid and dehydrofusaric acid [3, 61], and/or into the infected *S. hermonthica* shoot to cause phytotoxicity, e.g., beauvericin [51]. Screening of the differential exometabolome of genetically contrasting *Fos* isolates to identify specific secondary metabolites with proven biopesticidal efficacy against *S. hermonthica* incidence *in planta* is lacking. This knowledge would bring awareness on specific bioactive metabolites that constitute the *Fos* exometabolomic arsenal for preventing *S. hermonthica* incidence.

The ability of several fungal secondary metabolites, especially *Fusarium* toxins, to hamper early growth stages (germination, germ tube development/attachment), and later stages (post-attachment/aboveground development) of the life cycle of parasitic weeds have been mentioned in numerous studies. With this, they have a considerable potential as biological tools for the parasitic weed management [10, 71, 77]. In the context of *S. hermonthica*, some specific *Fusarium* extracellular metabolites (exometabolites) have shown complete inhibition of the seed germination in vitro, when used as post-*Striga* seed conditioning treatments at very low concentrations (≤ 1 mM). For example, T-2 toxin at 10 μM [83], neosolaniol at 240 μM [67], and fusaric acid at 1 mM [28]. However, it is unknown if these highly bioactive *Fusarium* exometabolites will maintain their potency against *S. hermonthica* incidence *in planta*. Also, in non-parasitic plants, some *Fusarium* exometabolites have similarly shown to cause complete germination inhibition at very low concentrations, for instance; 1,4-naphthoquinone at 63 μM against *Pinus thunbergii* Parl. pollen germination [36], and hymeglusin at 50 μM against *Brassica juncea* (L.) Czern. seed germination [40]. For other *Fusarium* exometabolites, such as equisetin, the concentration causing complete inhibition of germination in plants has not been determined [78]. Unfortunately, the potential of these latter *Fusarium* exometabolites to inhibit *S. hermonthica* seed germination or incidence is not known. Thus, the identification of highly bioactive *Fusarium* exometabolites with broadscale efficacy against diverse *S. hermonthica* populations has been suggested as a promising direction for overcoming the limitations of the inconsistent effectiveness of *Fos* isolates against *S. hermonthica* [6]. In fact, fungal exometabolites, especially mycotoxins, play important roles in shaping the soil microbial community dynamics. This could impact the microbial abundance positively e.g., by serving as carbon source to support soil microbial proliferation; or adversely (antibiosis) e.g., by acting as antimicrobials to suppress soil microbial growth [44, 75]. Hence, in the latter case, microbial elimination (degradation) of the mycotoxin from the environment becomes a crucial ecological concern, as soil microorganisms are an integral part of any balanced ecosystem.

In spite of the progress made in identifying some particular *Fusarium* exometabolites that are effective at very low concentrations against *S. hermonthica* seed germination, there are still major knowledge gaps, especially regarding the exometabolite consistency in preventing *S. hermonthica* incidence *in planta*. Therefore, the main research questions of this study were: Among a set of known highly phytotoxic, germination-inhibiting *Fusarium* exometabolites [36, 40, 77], which is the most potent/efficient candidate (i.e., having the greatest effect at minimal concentration) against *S. hermonthica* seed germination? Is the potency of this candidate exometabolite consistent against *S. hermonthica* incidence *in planta*, whilst unaffecting the host crop development/yield? These questions serve as fundamental determiner for drawing greater attention into this strategy and encourage the use of fungal exometabolites as biopesticides to better combat the *S. hermonthica* menace. The study hypothesized that among the tested *Fusarium* exometabolites*,* the most potent/efficient candidate exometabolite against *S. hermonthica*, is part of the *Fos* exometabolome composition which is used for attacking *S. hermonthica*. Thus, the main objectives of the study were to: (i) examine the performance of a set of highly phytotoxic *Fusarium* exometabolites against *S. hermonthica* seed germination (in vitro) and incidence (*in planta*), towards identifying the most potent/efficient candidate exometabolite; and (ii) screen the exometabolome of contrasting *Fos* isolates (Foxy-2 and FK3), in determining if these agents produce the candidate exometabolite.

## Methods

### Preparatory activities: seed materials, seed sterilization, microbial samples, and exometabolites

*Striga hermonthica* (Delile) Benth. seeds were sampled from various locations in SSA (Table 1). Sorghum (*Sorghum bicolor* L. Moench) seeds (cultivar PI563294) were sampled from Maradi, Niger. Our previous research studies confirmed the susceptibility of this sorghum cultivar to *S. hermonthica* [5, 6]. The *S. hermonthica* and sorghum seeds were separately surface-sterilized using sodium hypochlorite, Tween 20® and double distilled water (ddH_2_O), then air-dried, according to the procedure of Anteyi and Rasche [5, 6]. *Fusarium oxysporum* f. sp. *strigae* (*Fos*) isolates Foxy-2 and FK3 were obtained from the University of Hohenheim, Stuttgart, Germany. *Fusarium venenatum* strain O86 (DSM number 23361) was obtained from the Leibniz Institute DSMZ (German Collection of Microorganisms and Cell Cultures) GmbH, Braunschweig, Germany. The following *Fusarium* exometabolites were purchased as isolated and purified forms from their suppliers; 1,4-naphthoquinone (1,4-Nq) (Sigma-Aldrich Chemie GmbH, Taufkirchen, Germany), diacetoxyscirpenol (DAS) (Romer Labs Deutschland GmbH, Butzbach, Germany), equisetin (Equi) (Sigma-Aldrich Chemie GmbH), fusaric acid (FuA) (Sigma-Aldrich Chemie GmbH), hymeglusin (Hym) (Sigma-Aldrich Chemie GmbH), neosolaniol (Neo) (VWR International GmbH, Bruchsal, Germany) and T-2 toxin (T-2) (BIOMOL GmbH, Hamburg, Germany).

**Table 1:** Sampled *S. hermonthica* origin and phenotypic response to *Fos* isolates Foxy-2 and FK3

***S. hermonthica*** origin	Geographic coordinates (in decimal degrees)		Source	Sampled population phenotypic response to ***Fos*** isolates Foxy-2 and FK3 ^a^
	Latitude	Longitude		
Abuja (Nigeria)	9.066667	7.483333	IITA-Nigeria	Susceptible to Foxy-2 and FK3
Wad-Medani (Sudan)	14.4	33.533333	UG-Sudan	Susceptible to Foxy-2 and FK3
Kibos (Kenya)	−0.066667	34.816667	CIMMYT-Kenya	Susceptible to FK3; non-susceptible to Foxy-2
Sirinka (Ethiopia)	11.75	39.6	SARC-Ethiopia	Susceptible to FK3; non-susceptible to Foxy-2

IITA-Nigeria – International Institute of Tropical Agriculture, Ibadan, Nigeria

UG-Sudan – University of Gezira, Wad Medani, Sudan

CIMMYT-Kenya – International Maize and Wheat Improvement Centre, Kibos research facility, Kenya

SARC-Ethiopia – Sirinka Agricultural Research Centre, Sirinka, Ethiopia

^a^ − according to [5]

### Performance of the *Fusarium* exometabolites against *S. hermonthica*

#### In vitro seed germination assay

The germination inhibitory potential of the test *Fusarium* exometabolites (i.e., 1,4-Nq, DAS, Equi, FuA, Hym, Neo and T-2) against *S. hermonthica* seeds were examined at 1, 5, 10, 20, 50 and 100 μM concentrations. The exometabolites were initially diluted with acetone (Carl Roth GmbH) to 1 mM, then by serial dilutions with ddH_2_O to the respective study concentrations. 100–250 surface-sterilized *S. hermonthica* seeds (collected from Sirinka, Ethiopia) were laid on a sterilized 0.8 cm glass fibre filter paper (GFFP) punched disc. Four *S. hermonthica* seed-carrying punched discs (i.e., four replicates) were placed on a doubled layer of sterilized 9 cm GFFP, contained in a 9 cm petri dish. Then, 6 mL of the exometabolites (or ddH_2_O as control) were added as pre-*Striga* seed conditioning treatments to each petri dish. Thus, the treatments included (1): ddH_2_O (control); (2): Hym; (3): 1,4-NQ; (4): FuA; (5): Equi; (6): Neo; (7): T-2; (8): DAS. For *S. hermonthica* seed preconditioning, the petri dishes were covered, sealed with parafilm and incubated for 14 d in a dark chamber at 30 °C. Afterwards, the GFFP punched discs (carrying the preconditioned *S. hermonthica* seeds) were re-placed on a single layer of new, sterilized 9 cm GFFP, in a petri dish. 3 mL of a synthetic strigolactone analog, rac-GR24 (0.1 ppm) (Chiralix B.V., Nijmegen, Netherlands) was added to every petri dish, and they were re-incubated at 30 °C for 24 h. This was followed by counting the germinated *S. hermonthica* seeds using the Zeiss Stemi 2000-C Stereomicroscope (Carl Zeiss Microscopy GmbH, Jena, Germany), together with the Zeiss AxioCam HRc (Carl Zeiss Light Microscopy, Göttingen, Germany). The experiment was performed in two repetitions. The least study concentration where all the tested exometabolites significantly reduced *S. hermonthica* seed germination was utilized as working concentration for further investigations.

#### Planting experiments

Sixty-five g of planting soil substrate, made up of a blend of 80% modular seed substrate (Klasmann-Deilmann GmbH, Geeste, Germany) and 20% maize rhizosphere (collected from the top layer (0–20 cm) of a maize field in Filderstadt, Germany: Latitude 48.6483, Longitude 9.2475) were filled into 20 × 5 × 2 cm rhizoboxes, made from polyvinyl chloride. Then a plexiglass lid was used to cover each of the rhizoboxes. Sixty-five mg of *S. hermonthica* seeds (from Sirinka, Ethiopia) were sown in depth of 5 cm beneath the soil surface. Thereafter, using a 10 mL pipette, each of the rhizoboxes were treated with 65 mL of the test exometabolites at 20 μM working concentration (derived from the in vitro seed germination assay), by application on the soil surface. Thus, the treatments included (1): ddH_2_O without *Striga* (*Striga*-neg. control); (2): ddH_2_O + *Striga* (*Striga*-pos. control); (3): Hym + *Striga*; (4): 1,4-Nq + *Striga*; (5): FuA + *Striga*; (6): Equi + *Striga*; (7): Neo + *Striga*; (8): T-2 + *Striga*; (9): DAS + *Striga*. Each treatment was replicated thrice, and the set up was arranged in a randomized complete block design. For *S. hermonthica* seed preconditioning, the rhizoboxes were incubated at 30 °C for 14 d in a climate chamber without light (Percival Intellus Environmental Controller, EA-75HIL, Perry, Iowa, USA). This was followed by sowing a sorghum seed in each of the rhizoboxes, and maintaining the climate chamber at an alternating 12-h period of 31 °C with light (mean illuminance 46,000 lx) and 27 °C without light. The rhizoboxes were irrigated with nutrient solution twice weekly, which consisted of a mixture of 3 parts of 0.2% (v/v) Wuxal® universal liquid fertilizer (Aglukon Spezialdünger GmbH, Düsseldorf, Germany) and 2 parts of Yoshida nutrient solution [79]. The planting experiments lasted for 9 weeks after sowing sorghum, and the trials were performed in 2 experimental repetitions. Following a similar experimental setup, additional planting experiments were performed, but without including *S. hermonthica* in the design. At the end of the planting experiments, *S. hermonthica* incidence in a rhizobox was determined from the total amount of attached *S. hermonthica* belowground and aboveground. The sorghum aboveground biomass was harvested, dried in an oven at 65 °C for 10 d, then weighed to obtain the sorghum aboveground dry biomass (ABM) data.

### Additional experiments focusing on DAS (the candidate exometabolite)

#### Consistency against diverse *S. hermonthica* populations

The germination inhibitory potential of DAS against seeds of *S. hermonthica* populations from Abuja (Nigeria), Wad-Medani (Sudan) and Kibos (Kenya) were also evaluated. The experiment followed similar technical procedures as described for the in vitro seed germination assay (see above). The treatments included (1): ddH_2_O (control); (2): DAS 1 μM; (3): DAS 20 μM. Each of the treatments were in 4 replications, and the experiment was done in two repetitions.

#### Effectiveness as a post-Striga seed conditioning treatment

The experiment followed similar technical procedures as described for the in vitro seed germination assay (see above). However, here, the 14-d *S. hermonthica* (from Sirinka, Ethiopia) seed preconditioning was performed with 6 mL ddH_2_O. Thereafter, 1.5 mL DAS (1 μM or 20 μM) was applied as a post-*Striga* seed conditioning treatment, together with 1.5 mL rac-GR24 (0.1 ppm). Hence, the treatments included **(1):** rac-GR24 (control); **(2):** DAS 1 μM + rac-GR24; **(3):** DAS 20 μM + rac-GR24. The treatments were in 3 replications each, and the experiment was done in two repetitions.

#### Impact on soil microbial community abundance

10 g of planting soil substrate were filled into 9 cm petri dishes, and supplied with either 10 mL of ddH_2_O, DAS 1 μM or DAS 20 μM. In a parallel setup, 10 mg unsterilized *S. hermonthica* seeds were mixed into the planting soil substrate in each of the petri dishes. Therefore, the treatments consisted of (1): ddH_2_O (*Striga*-neg. control); (2): ddH_2_O + *Striga* (*Striga*-pos. control); (3): DAS 1 μM; (4): DAS 1 μM + *Striga*; (5): DAS 20 μM; (6): DAS 20 μM + *Striga*. The treatments were in 3 replications each. Then, the petri dishes were covered and incubated in a dark chamber at 28 °C for 14 d. Thereafter, total DNA (deoxyribonucleic acid) was isolated from 500 mg of the incubated treatments using the Fast DNA® spin kit for soil (MP Biomedicals LLC, Solon, Ohio, USA). Effect of DAS on the soil microbial community abundance was evaluated from the major soil microbial groups i.e., bacteria and fungi, through the 16S ribosomal RNA (rRNA) and 18S rRNA gene copy numbers, respectively. This was done by real-time quantitative PCR (qPCR) on the StepOnePlus™ Real-Time PCR System (Applied Biosystems, Waltham, Massachusetts, USA). At a standardized DNA concentration of 10 ng μl^− 1^ for each treatment, a 20 μL qPCR reaction system, with 3-step cycling, was performed using the SensiFAST™ SYBR® Hi-ROX Kit (Bioline Meridian Bioscience, Paris, France), according to the manufacturer’s protocol. The primer information is provided in Table 2. qPCR runs for each treatment consisted of 3 biological replicates into 3 technical replicates.

**Table 2:** List of primers sets used for qPCR and RT-PCR analyses

Primer id	Nucleotide sequence (5′ → 3′)	Target gene	Target group	Reaction	Reference
Eub338 (F)	ACCTACGGGAGGCAGCAG	16S rRNA	Bacteria	qPCR	[39]
Eub518 (R)	ATTACCGCGGCTGCTGG	16S rRNA	Bacteria	qPCR	[50]
FF390 (F)	CGATAACGAACGAGACCT	18S rRNA	Fungi	qPCR	[73]
FR1 (R)	AICCATTCAATCGGTAIT	18S rRNA	Fungi	qPCR	[73]
FsTri5 (F)	TGGAGAACTGGATGGTCTGG	Tri5	TpFs	RT-qPCR	[2]
FsTri5 (R)	GACATAGCCGTGCATGAAGC	Tri5	TpFs	RT-qPCR	[2]
FsTri4 (F)	GCCACTGCTGCTACTGTTGA	Tri4	TpFs	RT-qPCR	[2]
FsTri4 (R)	GGTCGTTGTCCAGATGTTCTTG	Tri4	TpFs	RT-qPCR	[2]
EF1A (F)	GGCTTTCACCGACTACCCTCCTCT	EF1A	Eukaryotes	RT-qPCR	[32]
EF1A (R)	ACTTCTCGACGGCCTTGATGACAC	EF1A	Eukaryotes	RT-qPCR	[32]

F – Forward primer

R – Reverse primer

TpFs – Trichothecene producing *Fusarium* species (especially characterized in *F. graminearum* and *F. sporotrichioides*)

#### Degradation of DAS in the planting soil substrate (targeted metabolomics)

For sample isolation, the left-overs of the soil microbial cultures (see above) were transferred to sterile 50 mL falcon tubes, and 10 mL ethyl acetate (Merck KGaA, Darmstadt, Germany) were added to each tube. The tubes were incubated in a dark shaker overnight, at 100 rpm and 27 °C. Thereafter, the tubes were centrifuged for 20 min, at 4750 rpm and 4 °C, using the Allegra® X-15R centrifuge (Beckman Coulter GmbH, Krefeld, Germany). The ethyl acetate phase was isolated and utilized for targeted metabolomic analysis by liquid chromatography–mass spectrometry (LC-MS) system. For this, 500 μL of the ethyl acetate phase were completely dried under N_2_, and resuspended with 150 μL methanol (VWR, Radnor, Pennsylvania, USA). Five μL of the solution were injected into the Agilent 1290 Infinity LC, coupled with the HPLC column ZORBAX Eclipse Plus C18, 95 Å, 1.8 μm, 2.1 × 50 mm, at 40 °C (Agilent Technologies GmbH, Waldbronn, Germany). Mobile phases included 0.2% formic acid (VWR) (solvent A), and acetonitrile (VWR) with 0.2% (v/v) formic acid (solvent B). At a flow rate of 0.3 mL min^− 1^, the ‘time – composition’ gradient program was set at 0.00(min) – B = 0.0%, 1.50(min) – B = 0.0%, 7.00(min) – B = 15.0%, 19.00(min) – B = 75.0%, 22.00(min) – B = 100.0%, 22.10(min) – B = 0.0%, and 23.10(min) – B = 0.0%. All solvents used were LC-MS grade. The purified fraction, and standard DAS, were measured by electrospray ionization and fourier transform mass spectrometry, using the Q Exactive™ Plus Orbitrap™ System (Thermo Fisher Scientific, Waltham, Massachusetts, USA). It made use of positive polarity mode, with 4.2 kV spray capillary. A desolvation temperature of 380 °C and stepped collision energy of 20, 60, and 110 V were maintained. The data dependent acquisition method was adopted, with a full MS scan of 70,000 resolution, followed by dd-MS^2^ (TOP 5) 140 to 1200 *m/z* of 17,500 resolution. From the obtained [M + H] ^+^, [M + Na] ^+^ and [M + NH_4_] ^+^ adducts, the more intense [M + NH_4_] ^+^ adduct was selected for further analysis. For the standard DAS, at a retention time of 11.17 min, it was measured at 384.201 *m/z*.

### Screening of *Fos* exometabolome for DAS production

#### Fungal culturing and metabolic footprinting

1.6 cm agar-mycelial plug from actively growing potato dextrose agar (PDA) (Carl Roth GmbH) cultures of *Fos* isolates (Foxy-2 and FK3), including *F. venenatum* (as control), were separately transferred to sterile 50 mL falcon tubes containing 35 mL of 25% potato dextrose broth (PDB) (Carl Roth GmbH). *Fusarium venenatum* was used as control because of its ability to produce DAS [46]. In a parallel setup, 35 mg unsterilized *S. hermonthica* seeds were added to each tube. Thus, the treatments included **(1):** Foxy-2; **(2):** Foxy-2 + *Striga*; **(3):** FK3; (**4):** FK3 + *Striga*; **(5):** *F. venenatum*; **(6):** *F. venenatum* + *Striga*. The treatments were in 3 replications each. Then, the tubes were incubated in a dark shaker for 2 weeks, at 65 rpm and 28 °C. Following a similar experimental design, Foxy-2, FK3 and *F. venenatum* were also cultured using Czapek-Dox broth containing 2% peptone (Carl Roth GmbH), with static incubation for 3 weeks at 22 °C, under 16 h light and 8 h dark conditions [63]. Thereafter, 10 mL ethyl acetate were added to every tube, and they were re-incubated overnight. Then, the tubes were centrifuged for 20 min, at 4750 rpm and 4 °C. The ethyl acetate phase of the treatments was isolated and used for exometabolomic analysis by LC-MS system as described above.

#### RNA isolation and gene expression

Total RNA (ribonucleic acid) was isolated from 100 mg hyphal biomass of actively growing PDA cultures of Foxy-2, FK3 and *F. venenatum* (ISOLATE II RNA Plant Kit, Bioline GmbH, Luckenwalde, Germany). Trichothecene gene expression by the fungal isolates were determined by quantitative reverse transcription PCR (RT-qPCR), using the StepOnePlus™ Real-Time PCR System. At a standardized RNA concentration of 10 ng μl^− 1^ for each fungal sample, a 20 μl RT-qPCR reaction system, with 3-step cycling, was performed using the SensiFAST™ SYBR® Hi-ROX One-Step Kit (Bioline Meridian Bioscience), according to the manufacturer’s protocol. Trichothecene genes, Tri5 and Tri4, were the genes of interest. These are the first genes involved in the *Fusarium* trichothecene biosynthetic pathway, from the starting compound farnesyl pyrophosphate. Tri5 encodes trichodiene synthase (which converts farnesyl pyrophosphate to trichodiene), while Tri4 encodes a cytochrome P450 monooxygenase (which converts trichodiene to isotrichotriol). Isotrichotriol is converted to isotrichodermol (i.e., the first trichothecene in the pathway) through a non-enzymatic process. In presence of other relevant Tri*-*genes (i.e., Tri101, Tri11, Tri3, Tri13 and Tri7, and Tri8), isotrichodermol is eventually converted to more complex trichothecene chemical species e.g., DAS [29, 35]. Thus, *Fusarium* strains lacking trichodiene synthase do not produce trichothecenes [46]. A housekeeping gene, Eukaryotic translation elongation factor 1 alpha (EF1A), was used as the reference gene [32]. The primer information is given in Table 2. RT-qPCR runs for each fungal sample consisted of 3 biological replicates into 3 technical replicates. Relative gene expression was calculated by the Pfaffl equation [56].

### Biostatistical analysis

Quantitative data from all the experiments in this study were tested by the analysis of variance (ANOVA), and Tukey’s range test (post hoc analysis), using SAS 9.4 (SAS Institute, Cary, North Carolina, USA). In presenting the Tukey’s range test of the *S. hermonthica* incidence and sorghum ABM from the planting experiments, the mean data were standardized (as percentage) in relation to the respective control treatments, without making alterations to the analyses output. Thus, the *S. hermonthica* incidence data were standardized relative to the ‘*Striga*-pos. Control’, and the sorghum ABM data were standardized relative to the **‘***Striga*-neg. Control’.

## Results

### The most potent/efficient candidate exometabolite against *S. hermonthica*

**Fig. 1:**
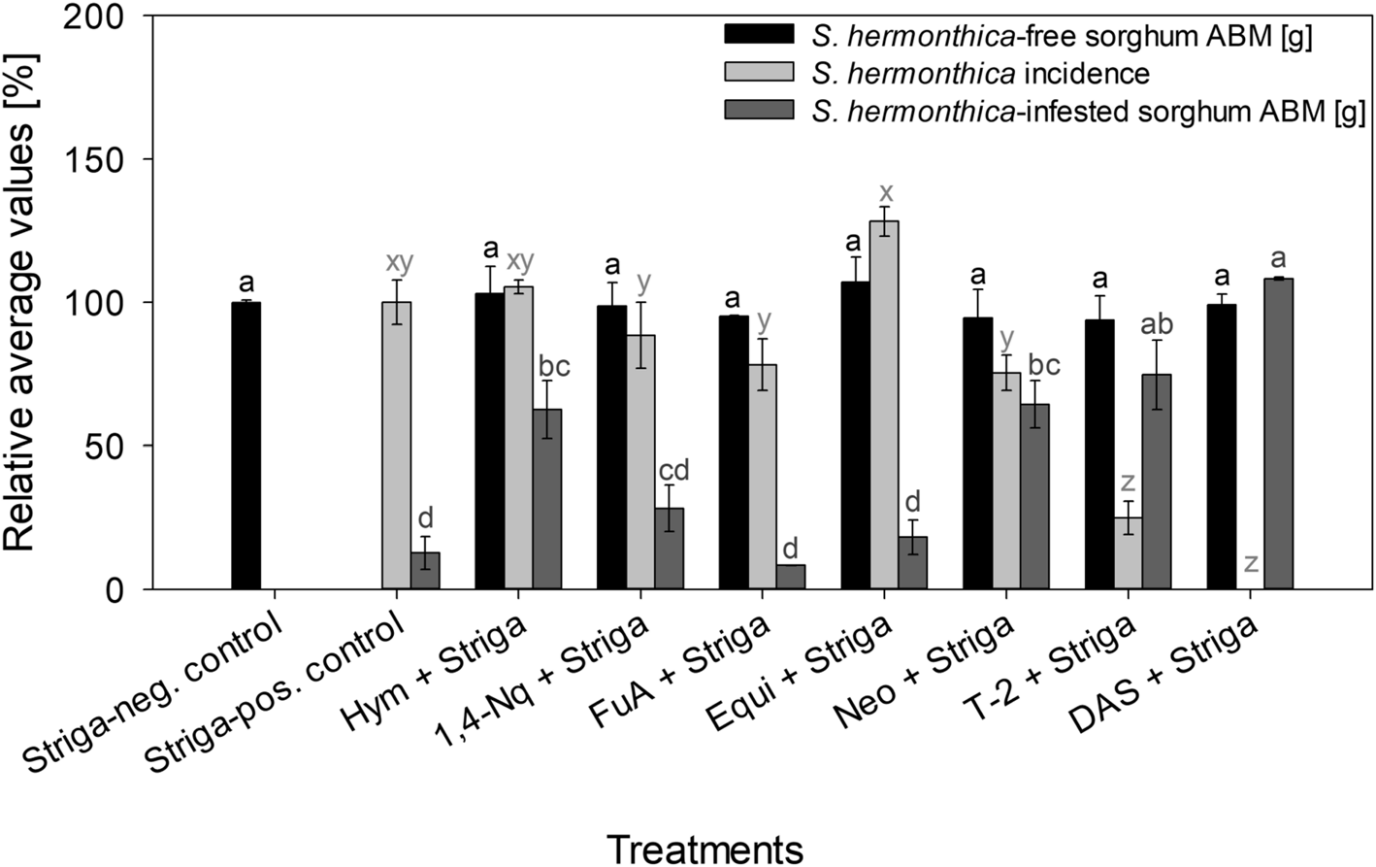
Relative average values of *S. hermonthica*-free sorghum ABM (aboveground dry biomass), *S. hermonthica *incidence and *S. hermonthica*-infested sorghum ABM to the chemical treatments at 20 μM concentration. *Striga*-neg. control = ddH_2_O without *S. hermonthica*. *Striga*-pos. control = ddH_2_O with *S. hermonthica*. Bars having at least a letter in common represent treatments means that are not significantly different (α = 0.05). Error bars indicate standard error (SE)

**Fig. 2:**
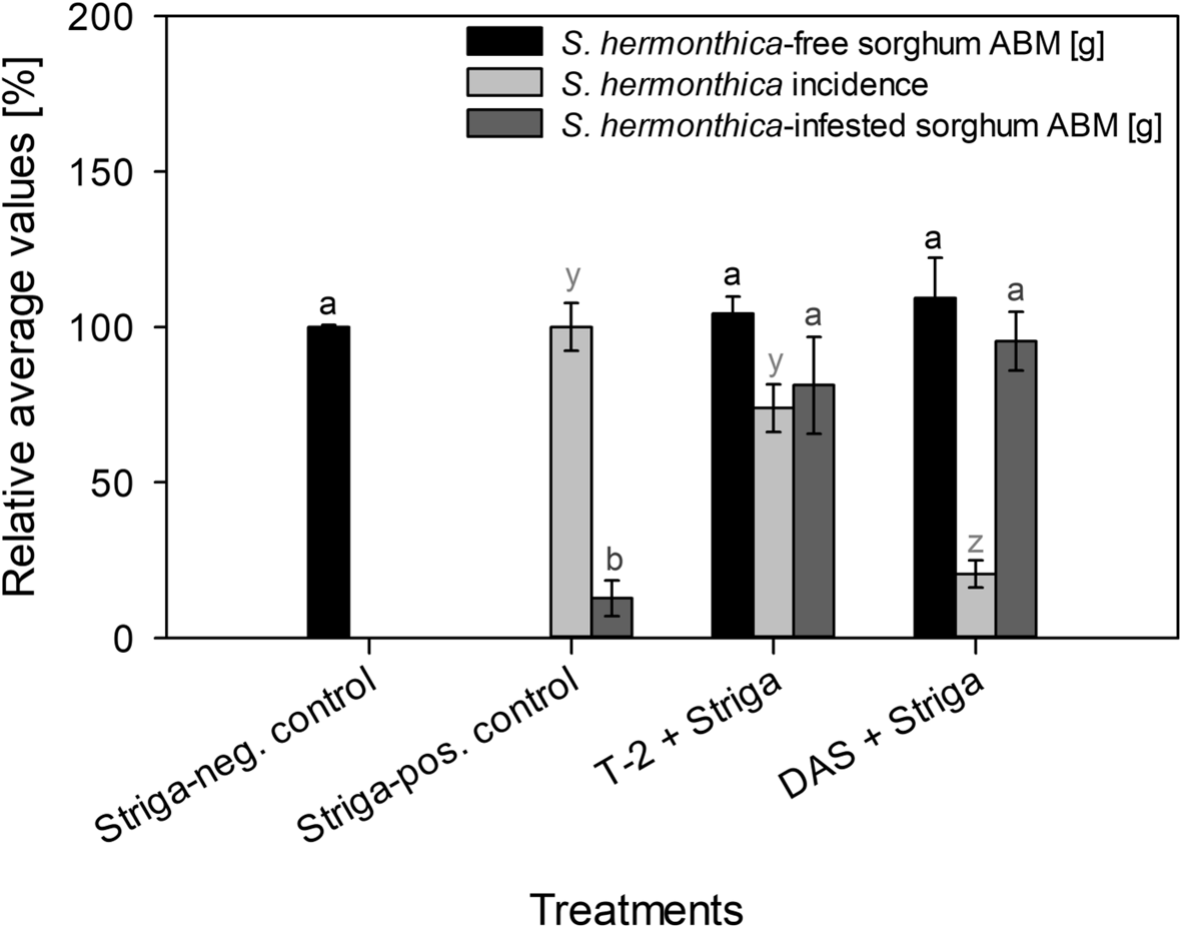
Relative average values of *S. hermonthica*-free sorghum ABM (aboveground dry biomass), *S. hermonthica *incidence and *S. hermonthica*-infested sorghum ABM to T-2 and DAS at 1 μM concentration. *Striga*-neg. control = ddH_2_O without *S. hermonthica*. *Striga*-pos. control = ddH_2_O with *S. hermonthica*. Bars having at least a letter in common represent treatments means that are not significantly different (α = 0.05). Error bars indicate standard error (SE)

ANOVA indicated that at the various study concentrations, the treatments caused different effect on *S. hermonthica* seed germination (*P* < 0.0001). Tukey’s range test further revealed that at 1–10 μM, some exometabolites, particularly DAS, T-2 and Neo, exhibited stronger *S. hermonthica* seed germination inhibition compared to the others (Table 3). This concentration range (1–10 μM) was also characterised by wide variability (standard error (SE) > 1) in most of the treatments effect, including high instability of the response (*S. hermonthica* seed germination inhibition) in relation to increasing treatment concentration. Nevertheless, as from 10 μM, inhibition of *S. hermonthica* seed germination followed a concentration-dependent trend in relation to increasing treatment concentration. At ≥20 μM, all the exometabolites significantly reduced *S. hermonthica* seed germination, and they displayed more accurate/stable treatment effects (SE < 1). Hence, 20 μM was utilized as the exometabolite treatments working concentration in the planting experiments. All exometabolites completely inhibited *S. hermonthica* seed germination at 100 μM. Out of all tested exometabolites, only DAS completely inhibited *S. hermonthica* seed germination at every concentration (1–100 μM). The planting experiments revealed that at 20 μM, none of the exometabolites affected the ABM of *S. hermonthica*-free sorghum (*P* > 0.05). However, only DAS and T-2 supressed *S. hermonthica* incidence (*P* < 0.0001) (Fig. 1). Correspondingly, except for DAS and T-2, the ABM of *S. hermonthica*-infested sorghum for the rest exometabolites was lower than the *Striga*-neg. Control (*P* < 0.0001). DAS, nonetheless, was outstanding compared to T-2, because DAS completely hindered *S. hermonthica* incidence. This resulted in a larger ABM of *S. hermonthica*-infested sorghum. Further evaluation of DAS and T-2 showed that their ability to prevent *S. hermonthica* incidence was lower at 1 μM (Fig. 2). Nevertheless, DAS still suppressed *S. hermonthica* incidence (*P* < 0.05), while *S. hermonthica* incidence for T-2 was not different from the *Striga*-pos. Control (*P* > 0.05). The ABM of *S. hermonthica*-infested sorghum for both exometabolites were not different to the *Striga*-neg. Control (*P* > 0.05).

**Table 3:** Tukey’s range test for *S. hermonthica* germination percentages (average) at different exometabolite concentrations

	1 μM	5 μM	10 μM	20 μM	50 μM	100 μM
ddH_2_O	55.000 (1.414) ^a^	55.000 (1.414) ^a^	55.000 (1.414) ^ab^	55.000 (1.414) ^a^	55.000 (1.414) ^a^	55.000 (1.414) ^a^
Hym	37.958 (4.495) ^ab^	40.798 (1.366) ^a^	11.362 (1.862) ^de^	0.551 (0.225) ^c^	0.689 (0.264) ^b^	0.000 (0.000) ^b^
1,4-NQ	32.148 (3.309) ^bc^	34.859 (6.083) ^ab^	37.958 (8.161) ^bc^	4.135 (0.574) ^b^	0.965 (0.347) ^b^	0.000 (0.000) ^b^
FuA	28.662 (2.569) ^bc^	35.117 (5.164) ^ab^	16.268 (8.521) ^de^	1.516 (0.823) ^bc^	0.000 (0.000) ^b^	0.000 (0.000) ^b^
Equi	15.106 (6.939) ^cd^	54.225 (6.512) ^a^	26.338 (0.894) ^cd^	0.551 (0.225) ^c^	0.000 (0.000) ^b^	0.000 (0.000) ^b^
Neo	3.486 (2.480) ^d^	5.423 (0.775) ^c^	0.775 (0.447) ^e^	0.000 (0.000) ^c^	0.000 (0.000) ^b^	0.000 (0.000) ^b^
T-2	4.648 (1.096) ^d^	0.775 (0.775) ^c^	0.000 (0.000) ^e^	0.000 (0.000) ^c^	0.000 (0.000) ^b^	0.000 (0.000) ^b^
DAS	0.000 (0.000) ^d^	0.000 (0.000) ^c^	0.000 (0.000) ^e^	0.000 (0.000) ^c^	0.000 (0.000) ^b^	0.000 (0.000) ^b^

Means having at least a letter in common are not significantly different (α = 0.05). Standard error (SE) is in parenthesis

**Fig. 3:**
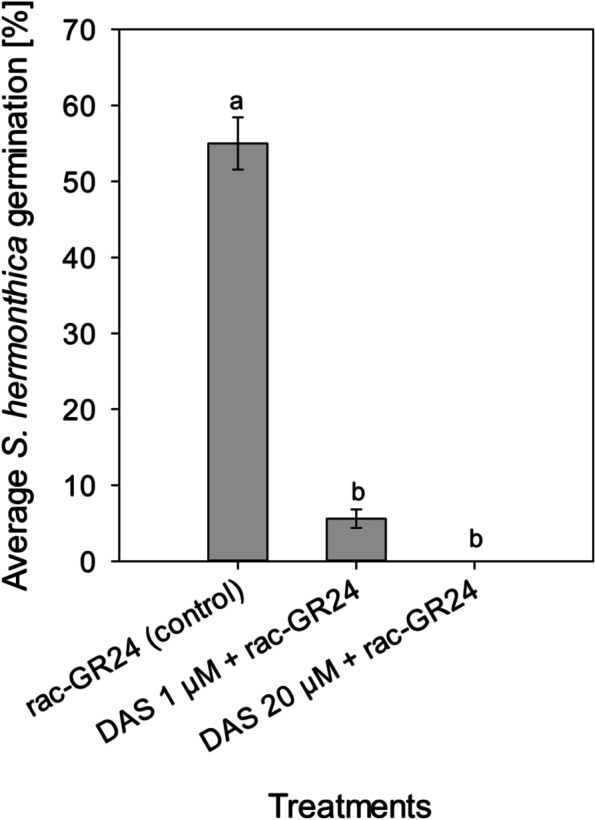
Average *S. hermonthica *germination response to post-*Striga *seed conditioning treatment with DAS*. *Bars having at least a letter in common represent treatments means that are not significantly different (α = 0.05). Error bars indicate standard error (SE)

**Fig. 4:**
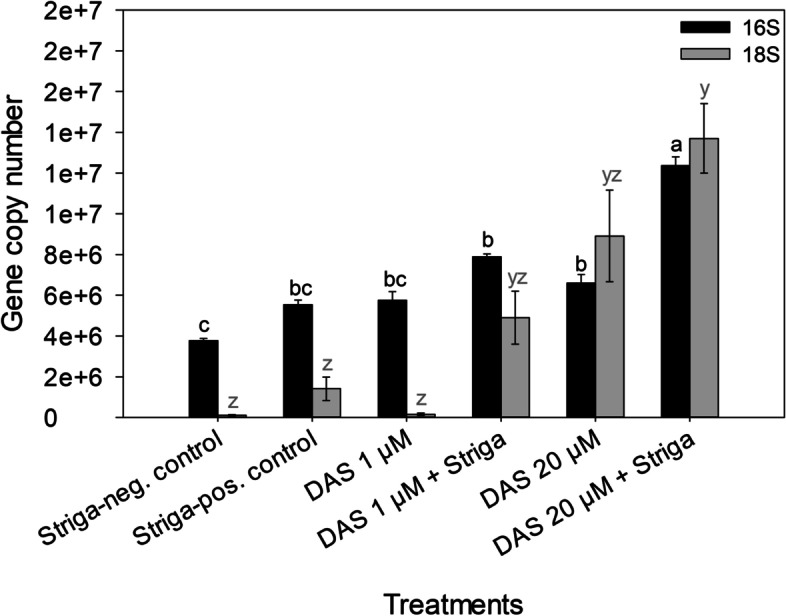
16S rRNA and 18S rRNA gene copy numbers in planting soil substrate after a 14-d incubation period*. Striga*-neg. control = soil + ddH_2_O without *S. hermonthica *seeds*. Striga*-pos. control = soil + ddH_2_O + *S. hermonthica *seeds. Bars having at least a letter in common represent treatments means that are not significantly different (α = 0.05). Error bars indicate standard error (SE)

**Fig. 5:**
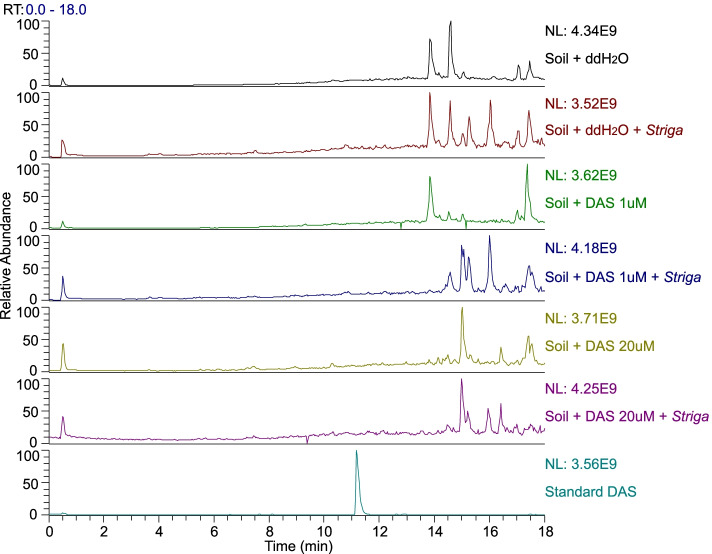
Mass chromatogram of planting soil substrate samples after a 14-d incubation. Standard DAS signal at 11.17 min

At 20 μM, DAS consistently inhibited the seed germination of diverse *S. hermonthica* populations from Abuja (Nigeria), Wad-Medani (Sudan) and Kibos (Kenya) completely (Table 4). Complete germination inhibition of the diverse *S. hermonthica* populations was also achieved at 1 μM DAS, except for the population from Kibos, which was almost completely inhibited (*P* < 0.0001). Similarly, application of DAS as a post-*Striga* seed conditioning treatment resulted in the complete inhibition of *S. hermonthica* seed germination at 20 μM (Fig. 3). This potential was reduced at 1 μM DAS, nevertheless, it was effective (*P *< 0.0001). Incubation of the planting soil substrate with 20 μM DAS for 14 days, strikingly increased the copy numbers of the 16S rRNA (bacterial community) (*P* < 0.0001) and the 18S rRNA (fungal community) genes (*P* < 0.01) (Fig. 4). The difference between the *Striga*-pos. and *Striga*-neg. control treatments indicated that addition of the *S. hermonthica* seeds moderately promoted the microbial abundance in the setup. This could have occurred either by contamination from the unsterilized *S. hermonthica* seeds, or the *S. hermonthica* seeds serving as nutrient substrate for microbial growth. However, comparing the DAS-treated samples to the control treatments revealed that DAS application stimulated proliferation of the bacterial and fungal communities in a concentration-dependent manner (i.e., 20 μM > 1 μM > control). Whereby, the fungal community multiplied faster than the bacterial community, as DAS concentration increased. LC-MS analysis revealed that DAS was eventually degraded in the planting soil substrate after the 14-d incubation. This was indicated by the absence of DAS signal in the LC-MS data of the DAS-treated samples (Fig. 5).

**Table 4:** Tukey’s range test for germination percentages (average) of diverse *S. hermonthica* populations at different DAS concentrations

	Abuja (Nigeria)	Wad-Medani (Sudan)	Kibos (Kenya)
ddH_2_O (control)	27.375 (2.625) ^a^	31.250 (1.750) ^a^	34.500 (0.833) ^a^
DAS 1 μM	0.000 (0.000) ^b^	0.000 (0.000) ^b^	0.583 (0.468) ^b^
DAS 20 μM	0.000 (0.000) ^b^	0.000 (0.000) ^b^	0.000 (0.000) ^b^

Means having at least a letter in common are not significantly different (α = 0.05). Standard error (SE) is in parenthesis

### Determination of DAS production by *Fos*

**Fig. 6:**
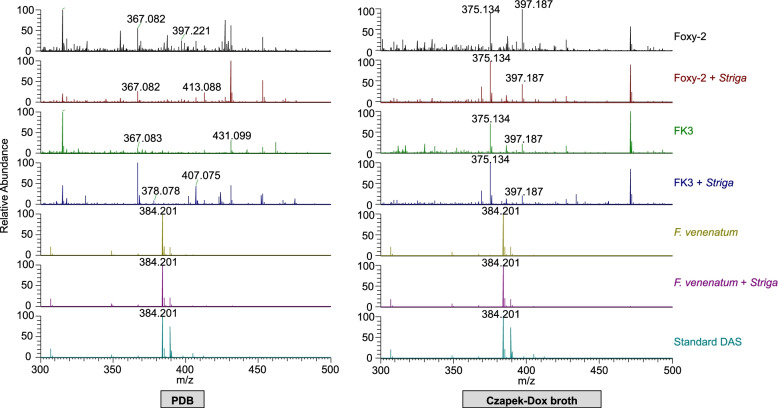
Mass spectrum of fungal samples cultured in PDB and Czapek-Dox broth media

**Fig. 7:**
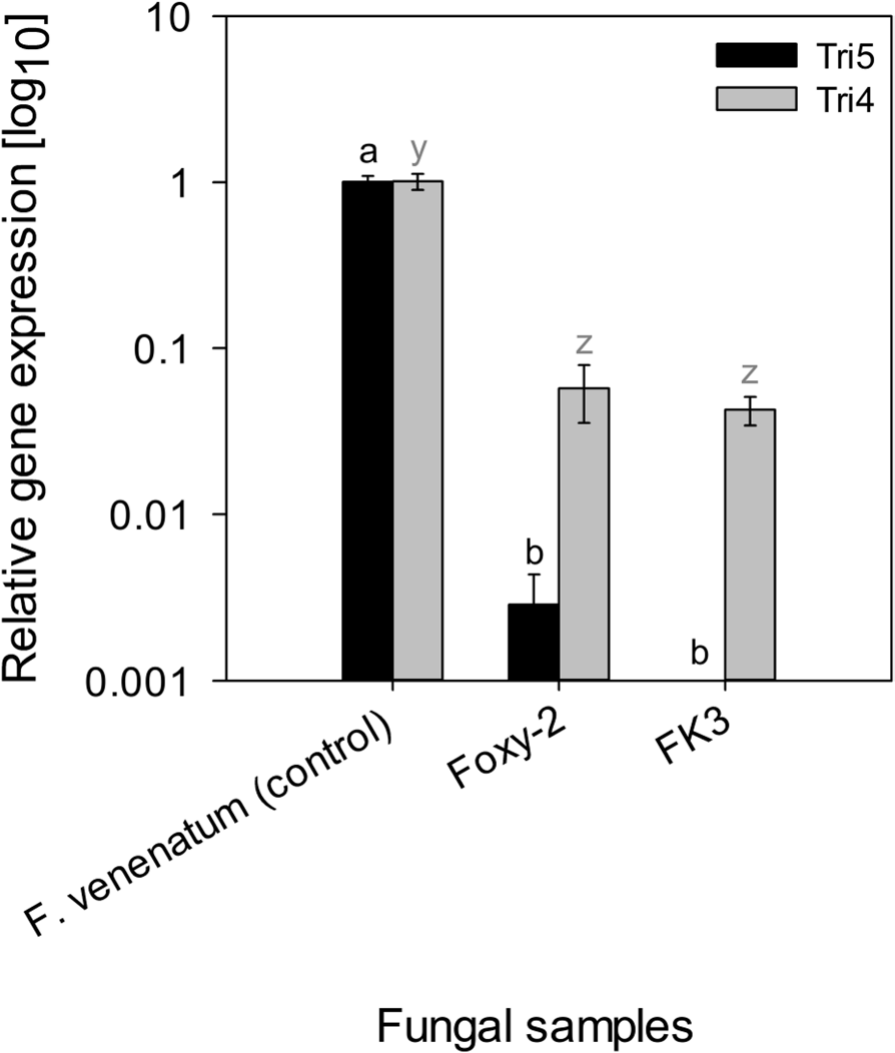
Relative expression of trichothecene genes Tri5 and Tri4 by *F. venenatum *and *Fos *isolates (Foxy-2 and FK3). Bars having at least a letter in common represent treatments means that are not significantly different (α = 0.05). Error bars indicate standard error (SE)

Exometabolomic investigation revealed that *F. venenatum* produced DAS in both the PDB and Czapek-Dox broth media cultures. This was regardless of the addition or non-addition of *S. hermonthica* seeds to the culturing media. Contrarily, both *Fos* isolates (Foxy-2, FK3) did not produce DAS under similar culturing conditions (Fig. 6). RT-qPCR analysis further revealed that for both Foxy-2 and FK3, the trichothecene genes Tri5 and Tri4 were underexpressed (*P* < 0.0001) (Fig. 7). *Fusarium venenatum* expressed Tri5 gene at 349.8-fold higher than Foxy-2, and 7951.3-fold higher than FK3. Also, *F. venenatum* expressed Tri4 gene at 17.7-fold higher than Foxy-2, and at 23.8-fold higher than FK3.

## Discussion

### DAS revealed outstanding suppression of *S. hermonthica*

It was the fundamental finding of this study that out of all tested *Fusarium* exometabolites, DAS exhibited the greatest potential to control *S. hermonthica*. DAS presented a clearly larger advantage than the other tested exometabolites in terms of its ability to completely inhibit *S. hermonthica* seed germination in vitro, at all studied concentrations (1–100 μM). Comparably, in a previous study on *Orobanche ramosa* (also a parasitic weed), DAS was the most promising *Fusarium* toxin tested as well, where it caused complete inhibition of the seed germination at 10 and 100 μM, but at 1 and 0.1 μM, it suppressed the germination to 2 and 7%, respectively [84]. With non-parasitic plants, on the other hand, 1 μM DAS failed to inhibit *Arabidopsis* seed germination, but at 10 μM, the germination was reduced from about 95 to 40% [42]. Furthermore, in *Nicotiana sylvestris*, pollen germination inhibition started from 20 ng mL^− 1^ (0.055 μM) DAS at 26.7%, but at 200 ng mL^− 1^ (0.55 μM), it was completely inhibited [65]. Because the variable susceptibility of diverse *S. hermonthica* populations to *Fos* isolates has been a major drawback in biocontrol of *S. hermonthica* [5, 7], the ability of DAS to consistently inhibit seed germination of diverse *S. hermonthica* populations in our study is a very interesting discovery. After DAS, T-2 was the second best-performing exometabolite tested in our study, followed by Neo. DAS, T-2 and Neo are all type A trichothecenes. Trichothecenes are sesquiterpene mycotoxins that are made up of a core ring structure, which contains an epoxide (tricyclic ether) at the 12,13 carbon positions, including a double bond at the 9, 10 carbon positions (see Supplementary File). These functional groups (particularly the former) are mainly responsible for their toxicity [14]. Also, type A trichothecenes differ from other trichothecene types i.e., type B (e.g., deoxynivalenol), type C (e.g., crotocin) and type D (e.g., satratoxin H), primarily because of the substitution at the carbon 8 of the core ring structure. This could be a hydroxyl group (e.g., Neo), an ester function (e.g., T-2), or no functional group/oxygen substitution (e.g., DAS) [45]. Hence, this group sameness may explain the moderately close biopesticidal potential of the type A trichothecenes against *S. hermonthica* in our study, particularly DAS and T-2. Nevertheless, T-2 performance was limited by several factors. For instance, it started causing complete germination inhibition of *S. hermonthica* seeds in vitro as from 10 μM (this was in agreement with Zonno and Vurro [83]), it was unable to completely hinder *S. hermonthica* incidence at 20 μM *in planta*, and it was unable to suppress *S. hermonthica* incidence at 1 μM. Moreover, due to loss of acetyl side groups on carbons 4 and 15, and an isovaleryl group at carbon 8, toxicity of DAS is generally lower [20, 42, 69]. We assume that the unique chemical structure of DAS may be the basis for its outstanding biopesticidal performance against *S. hermonthica*, compared to all other exometabolites tested in this study.

Preconditioning (i.e., dark stratification over a given period, in presence of moisture and suitably warm-temperature), is an indispensable, preliminary treatment, that is required for the release of parasitic plant seeds (*Orobanche* sp., *Striga* sp.) from primary dormancy, in preparation for germination [43]. During the seed preconditioning stage, essential metabolic pathways in the seed, such as respiration, synthesis of DNA, protein and hormones, are activated [17, 30]. Previous studies had mainly focused on using fungal exometabolites as post-*Striga* seed conditioning treatment against a single *S. hermonthica* population, mostly sampled from Sudan [1, 28, 67, 83]. However, as shown from our study, pre-*Striga* seed conditioning treatment with DAS consistently inhibited seed germination of diverse *S. hermonthica* populations, and DAS was effective both as a pre- and post-*Striga* seed conditioning treatment. It therefore implied that the mode by which DAS specifically inhibited *S. hermonthica* seed germination is not limited to the general disruption of vital cytological pathways or processes that occurs during *S. hermonthica* seed preconditioning for which trichothecenes are widely known, such as disruption of protein/DNA synthesis, mitochondrial function and mitotic division [9, 15, 24]. Perhaps, it could be connected to the disruption of other post-*Striga* seed conditioning pathways that are essential for *S. hermonthica* seed germination e.g., strigolactone perception. Because the strigolactone signal, which triggers *S. hermonthica* seed germination is perceived by strigolactone receptor, hence disruption of the strigolactone receptor (e.g., by mutation, or physical/chemical inhibitors) could lead to the inability of the receptor to perceive/transduce strigolactone signal [60, 62]. For instance, certain chemical inhibitors of the strigolactone receptors in some flowering plants (i.e., the α/β-hydrolase family proteins e.g., decreased apical dominance *2* in petunia (*DAD2*), and DWARF14 (*D14*) in *Arabidopsis* (*AtD14*) and rice (*OsD14*)), were recently identified. Examples of these inhibitors include, 2MN [41], N-Ph [25] and DL1 [80]. Thus, this major variation in the *S. hermonthica* seed germination pathway may serve as an important point of action for DAS. However, the exact mode of DAS inhibitory action against *S. hermonthica* seed germination remained elusive.

### DAS application *in planta* did not affect sorghum biomass and soil microbial abundance

Treatment of the planting soil substrate with 20 μM DAS did not affect the ABM of *S. hermonthica*-free sorghum. This observation was the same for all tested exometabolites. It was previously reported that shoot morphogenesis was severely inhibited in *Nicotiana tabacum* grown on DAS-treated MS agar as from 5 μM, but was completely blocked at 20 μM DAS [49]. Also, *Arabidopsis* shoot growth was severely inhibited and root elongation completely blocked, when grown on 10 μM DAS-treated MS agar [42]. Thus, the extent of toxicity of DAS and other trichothecenes varies with the test plant [42, 55]. This, therefore, corroborates the need to ascertain an exometabolite working concentration on *S. hermonthica* host plants, before the exometabolite is adopted for parasitic weed management.

The soil bacterial and fungal community abundances were increased in relation to increasing DAS concentration applied to the planting soil substrate. Accordingly, this observation coincided with the complete degradation of DAS in the soil substrates after a 14-d incubation of the experimental setup. Numerous microbes, especially bacteria and fungi, possess enzymatic gene products to specifically and efficiently degrade the 12,13-epoxy ring that is important for trichothecene toxicity [16, 74]. For instance, bovine rumen microorganisms e.g., *Eubacteria* strain BBSH 797, selectively degrade the 12,13-epoxy ring of DAS, T-2 and deoxynivalenol by enzymatic reduction into de-epoxy and deacylated products [21, 68]. Also, widely diverse soil and freshwater bacteria species including, but not limited to, *Curtobacterium* sp., *Bacillus* sp., *Pseudomonas* sp., *Arthrobacter* sp. *Agrobacterium* sp., *Brevibacterium* sp., *Blastobacter* sp., *Alcaligenes* sp., *Microbacterium* sp. and *Xanthomonas* sp., have been reported to completely degrade T-2, DAS, Neo and type B trichothecenes (nivalenol and fusarenon-X); whereby, the degraded product is assimilated and utilized as a source of carbon and energy for cellular growth/multiplication [8, 72]. On the other hand, fungal degradation of trichothecenes is mainly by acetylation and deacetylation of the trichothecene molecule to produce significantly less-toxic acetoxy products, without necessarily affecting the trichothecene core [81]. For example, the degradation of DAS by resting cells of *Mucor mucedo* and growing cells of *F. oxysporum* f. sp. *vasinfectum*, to produce triacetoxyscirpene and 3-acetoxyscirpene-4,15-diol respectively [11, 12]. Thus, the ability of numerous environmental microbes to efficiently degrade trichothecenes, and assimilate the end products as carbon/energy source for cellular multiplication, may explain the correlation we observed between increasing DAS treatment concentration in the planting soil substrate and the soil microbial community abundance, whereby DAS was eventually degraded in the planting soil substrate.

### *Fos* does not produce DAS

*Fos*, represented by Foxy-2 and FK3 isolates, did not produce DAS according to the exometabolomic (LC-MS) analysis. This observation corresponded to both *Fos* isolates underexpression of key genes (Tri5 and Tri4) that are necessary for initiating the *Fusarium* trichothecene biosynthesis pathway. It was on this note that the expression of Tri101, Tri11, Tri3, Tri13 and Tri7, and Tri8 genes by *Fos* were not further analysed in this study, since Foxy-2 and FK3 showed underexpression of Tri5 and Tri4 genes; an observation in line with the *Fos* exometabolomic analysis. This discovery gives a new insight that DAS (or essentially, trichothecenes) is not part of *Fos* exometabolome composition. This finding was in accordance with Proctor et al. [57] and Khatibi et al. [31], that categorized *F. oxysporum* as a non-producer of trichothecenes. However, it is worthy to note that some authors had reported the isolation of trichothecenes e.g., DAS and T-2, from *F. oxysporum* [22, 27, 47]. Although, Ghosal et al. [22] had clearly specified the *forma specialis* of the *F. oxysporum* they investigated i.e., *F. oxysporum* f. sp. *carthami*, which was different from ours. However, Mirocha et al. [47] and Hasan [27] did not further specify the *forma specialis* of *F. oxysporum* used in their experiment. It was revealed that *F. oxysporum* carry both non-functional (pseudo-Tri101) and functional (Tri201) trichothecene 3-*O*-acetyltransferase genes in their genome. The Tri201 gene acts like the Tri101 gene in known *Fusarium* trichothecene producers (e.g., *F. sporotrichioides*), which is responsible for C3 acetylation of 3-hydroxytrichothecenes (e.g., isotrichodermol) into a less-toxic product (e.g., isotrichodermin). This action is both a means of self-defence against toxic trichothecenes, and is also part of the *Fusarium* trichothecene biosynthesis pathway [33–35]. Therefore, it might be suggested that the ancestor of *F. oxysporum* was a trichothecene producer, before the divergence of trichothecene producers from non-producers in the evolution of *Fusarium* species [70]. Our finding, in contrast to Ghosal et al. [22], Mirocha et al. [47], Hasan [27], perhaps illustrates the proposed divergent evolution of trichothecene producers from non-producers in *F. oxysporum*.

## Conclusion

Among the tested *Fusarium* exometabolites, DAS was unequalled for completely inhibiting *S. hermonthica* seed germination in vitro, and preventing *S. hermonthica* incidence *in planta*. Notwithstanding the promising attributes of DAS in this study, there is need to further investigate its specific mode of action against the germination of *S. hermonthica* seeds. This investigation would be a critical step, before performing in situ verification (field trials) of the *S. hermonthica*-biopesticidal efficacy of DAS. Through this, DAS specificity of action against the target weed (*S. hermonthica*) at very low concentrations (≤ 20 μM), as opposed to non-target soil organisms, will be clearly understood.

Also, our study revealed that contrary to *F. venenatum, Fos* is a non-producer of DAS. It therefore raises the question if *F. venenatum* could be a complementary bioherbicide for controlling *S. hermonthica*. In this regard, an additional positive feature of this ubiquitous soil-borne saprophytic fungus is its inability to produce some mycotoxins, including T-2, deoxynivalenol, nivalenol, zearalenone and sambucoin [46]. Furthermore, *F. venenatum* is phylogenetically closely related to the phytopathogenic fungi *F. graminearum*, which is globally notorious for causing Fusarium head blight in cereals and vascular wilt to non-cereal plants, however, the non-phytopathogenic status of *F. venenatum* was confirmed [37]. Thus, as part of future research directions, *F. venenatum* could be tested for its *S. hermonthica* incidence prevention *in planta*. This will reveal whether the quantity of DAS produced by *F. venenatum* in the soil will sufficiently prevent *S. hermonthica* germination, whilst unaffecting non-target organisms. Therefore, *F. venenatum* could serve as a sustainable, cheaper (compared to isolated/purified DAS), and proactive biocontrol agent for *S. hermonthica* eradication. Another option would be to test if the co-inoculation of *F. venenatum* and *Fos* isolate (with known pathogenicity towards the given *S. hermonthica* population) will better increase the overall *S. hermonthica* biocontrol efficiency through synergism. This is based on the assumption that DAS from *F. venenatum* will primarily attack *S. hermonthica* germination, while *Fos* will attack the incidence of germinated or attached *S. hermonthica* seedlings that escaped the reach of DAS in the soil.

## Supplementary Information

**Additional file 1.**

## Data Availability

All data supporting the findings in this study are presented within the manuscript.

## Abbreviations

1,4-Nq: 1,4-naphthoquinone
ABM: Aboveground dry biomass
ANOVA: Analysis of variance
CIMMYT-Kenya: International Maize and Wheat Improvement Centre, Kibos research facility, Kenya
DAS: Diacetoxyscirpenol
DNA: Deoxyribonucleic acid
EF1A: Eukaryotic translation elongation factor 1 alpha
Equi: Equisetin
F: Forward primer
*Fos*: *Fusarium oxysporum* f. sp. *strigae*
FuA: Fusaric acid
Hym: Hymeglusin
IITA-Nigeria: International Institute of Tropical Agriculture, Ibadan, Nigeria
LC-MS: Liquid chromatography-mass spectrometry
Neo: Neosolaniol
PDA: Potato dextrose agar
PDB: Potato dextrose broth
qPCR: Real-time quantitative Polymerase chain reaction
R: Reverse primer
RNA: Ribonucleic acid
rRNA: Ribosomal ribonucleic acid
RT-qPCR: Quantitative reverse transcription Polymerase chain reaction
SARC-Ethiopia: Sirinka Agricultural Research Centre, Sirinka, Ethiopia
SE: Standard error
SSA: Sub-Saharan Africa
T-2: T-2 toxin
TpFs: Trichothecene producing *Fusarium* species
UG-Sudan: University of Gezira, Wad Medani, Sudan
