# Supplementary material for: Diacetoxyscirpenol, a Fusarium exometabolite, prevents efficiently the incidence of the parasitic weed Striga hermonthica
Source: BMC Plant Biol. 2022 Feb 24;22:84. doi: 10.1186/s12870-022-03471-6 (PMC8867772; doi:10.1186/s12870-022-03471-6)
Supplement: Supplementary file 1 — Additional file 1. [file 12870_2022_3471_MOESM1_ESM.pptx]

## Slide 1
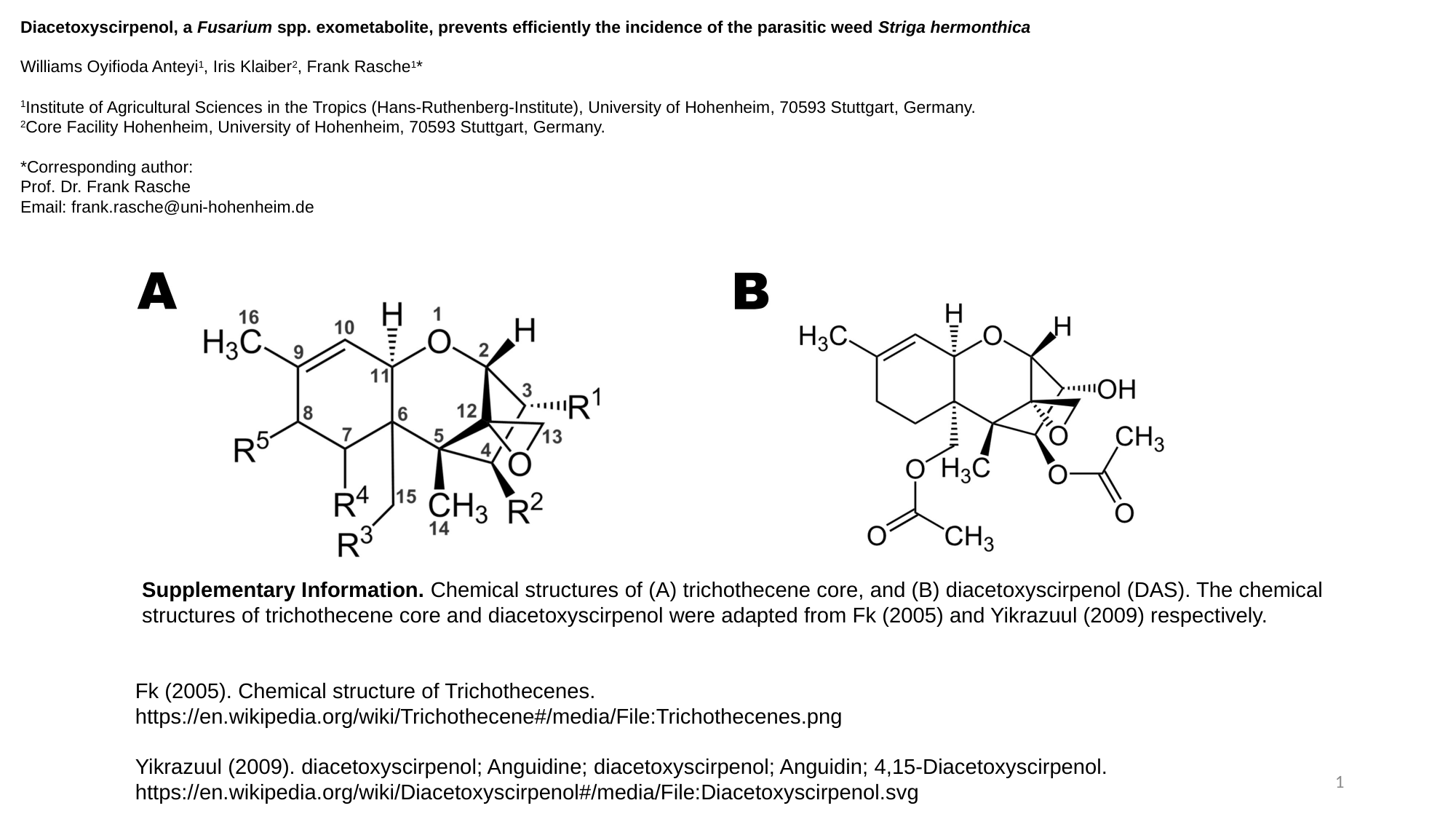

Diacetoxyscirpenol, a Fusarium spp. exometabolite, prevents efficiently the incidence of the parasitic weed Striga hermonthica
Williams Oyifioda Anteyi1, Iris Klaiber2, Frank Rasche1*
1Institute of Agricultural Sciences in the Tropics (Hans-Ruthenberg-Institute), University of Hohenheim, 70593 Stuttgart, Germany.
2Core Facility Hohenheim, University of Hohenheim, 70593 Stuttgart, Germany.
*Corresponding author:
Prof. Dr. Frank Rasche
Email: frank.rasche@uni-hohenheim.de
Supplementary Information. Chemical structures of (A) trichothecene core, and (B) diacetoxyscirpenol (DAS). The chemical structures of trichothecene core and diacetoxyscirpenol were adapted from Fk (2005) and Yikrazuul (2009) respectively.
Fk (2005). Chemical structure of Trichothecenes.
https://en.wikipedia.org/wiki/Trichothecene#/media/File:Trichothecenes.png
Yikrazuul (2009). diacetoxyscirpenol; Anguidine; diacetoxyscirpenol; Anguidin; 4,15-Diacetoxyscirpenol.
https://en.wikipedia.org/wiki/Diacetoxyscirpenol#/media/File:Diacetoxyscirpenol.svg
1
